# Local Delivery of High-Dose Chondroitinase ABC in the Sub-Acute Stage Promotes Axonal Outgrowth and Functional Recovery after Complete Spinal Cord Transection

**DOI:** 10.1371/journal.pone.0138705

**Published:** 2015-09-22

**Authors:** Chu-Hsun Cheng, Chi-Te Lin, Meng-Jen Lee, May-Jywan Tsai, Wen-Hung Huang, Ming-Chao Huang, Yi-Lo Lin, Ching-Jung Chen, Wen-Cheng Huang, Henrich Cheng

**Affiliations:** 1 Program in Molecular Medicine, National Yang-Ming University and Academia Sinica, Taipei, Taiwan; 2 Institute of Pharmacology, School of Medicine, National Yang-Ming University, Taipei, Taiwan; 3 Neural Regeneration Laboratory, Department of Neurosurgery, Neurological Institute, Taipei Veterans General Hospital, Taipei, Taiwan; 4 Center for Neural Regeneration, Neurological Institute, Taipei Veterans General Hospital, Taipei, Taiwan; 5 Department of Neurosurgery, Neurological Institute, Taipei Veterans General Hospital, Taipei, Taiwan; 6 Department of Medicine, National Yang-Ming University, Taipei, Taiwan; 7 Division of Pediatric Neurosurgery, Neurological Institute, Taipei Veterans General Hospital, Taipei, Taiwan; 8 School of Medicine, Taipei Medical University, Taipei, Taiwan; 9 Graduate Institute of Veterinary Pathobiology, College of Veterinary Medicine, National Chung Hsing University, Taichung, Taiwan; 10 Department of Applied Chemistry, Chaoyang University of Technology, Taichung, Taiwan; 11 Department of Nursing, Central Taiwan University of Science and Technology, Taichung, Taiwan; Hertie Institute for Clinical Brain Research, University of Tuebingen., GERMANY

## Abstract

Chondroitin sulfate proteoglycans (CSPGs) are glial scar-associated molecules considered axonal regeneration inhibitors and can be digested by chondroitinase ABC (ChABC) to promote axonal regeneration after spinal cord injury (SCI). We previously demonstrated that intrathecal delivery of low-dose ChABC (1 U) in the acute stage of SCI promoted axonal regrowth and functional recovery. In this study, high-dose ChABC (50 U) introduced via intrathecal delivery induced subarachnoid hemorrhage and death within 48 h. However, most SCI patients are treated in the sub-acute or chronic stages, when the dense glial scar has formed and is minimally digested by intrathecal delivery of ChABC at the injury site. The present study investigated whether intraparenchymal delivery of ChABC in the sub-acute stage of complete spinal cord transection would promote axonal outgrowth and improve functional recovery. We observed no functional recovery following the low-dose ChABC (1 U or 5 U) treatments. Furthermore, animals treated with high-dose ChABC (50 U or 100 U) showed decreased CSPGs levels. The extent and area of the lesion were also dramatically decreased after ChABC treatment. The outgrowth of the regenerating axons was significantly increased, and some partially crossed the lesion site in the ChABC-treated groups. In addition, retrograde Fluoro-Gold (FG) labeling showed that the outgrowing axons could cross the lesion site and reach several brain stem nuclei involved in sensory and motor functions. The Basso, Beattie and Bresnahan (BBB) open field locomotor scores revealed that the ChABC treatment significantly improved functional recovery compared to the control group at eight weeks after treatment. Our study demonstrates that high-dose ChABC treatment in the sub-acute stage of SCI effectively improves glial scar digestion by reducing the lesion size and increasing axonal regrowth to the related functional nuclei, which promotes locomotor recovery. Thus, our results will aid in the treatment of spinal cord injury.

## Introduction

Traumatic spinal cord injury (SCI) leads to a loss of sensory and motor function because the damaged axons are unable to regrow. After SCI, a cascade of axonal regeneration inhibitors accumulate at the injury site, including myelin-derived proteins such as myelin-associated glycoprotein (MAG) [1], Nogo-A [2], and oligodendrocyte-myelin glycoprotein (OMgp) [3]; extracellular matrix-derived factors such as repulsive guidance molecules (RGMs), i.e., ephrins and semaphorins [4]; and the reactive astrocyte-derived extracellular matrix molecules chondroitin sulfate proteoglycans (CSPGs) [5, 6], which are the main components of the astroglial scar. By eliminating these inhibitors, we may enhance axonal outgrowth and functional recovery after SCI [7].

Glial scars formed by activated astrocytes are a prominent feature of CNS trauma, which has been well documented for many years. The inhibition of axonal regeneration by the glial scar is widely viewed as detrimental to clinical outcomes [8, 9]. Although many studies have investigated the fundamental aspects of the cellular and molecular mechanisms of glial scarring, effective therapeutic approaches for clinical management are lacking. After SCI, various CSPGs, such as aggrecan, neurocan, versican and NG2 [6], are densely deposited in the lesion site and contribute to scar formation, which is a major obstacle for the regeneration of the injured spinal cord. The molecules of the CSPG family share two common structures: a major core protein and a variable number of highly sulfated glycosaminoglycan (GAG) side chains. During embryonic development, CSPGs are important for guiding neurite formation and elongation [10–12]. In adults, these molecules play a role in stabilizing neuronal structures and synaptic connections and restricting neuroplasticity [13, 14]. After central nervous system (CNS) injury, glial scar formation contributes to both a physical and molecular barrier at the injury site to prevent axonal regrowth and reconnection. CSPGs, the major components of glial scars, have been shown to prevent axonal reconnection. CSPGs are upregulated from multiple cellular sources, including astrocytes (a major cellular source of glial scarring), oligodendrocytes and proliferating fibroblasts (a major cellular source of fibrotic scarring), after CNS injury. GAG side chains are the major factors responsible for blocking axon regrowth, and digestion of the GAG side chains by ChABC enhances axonal outgrowth and promotes functional recovery in various animal models [15–17].

**Fig 1:**
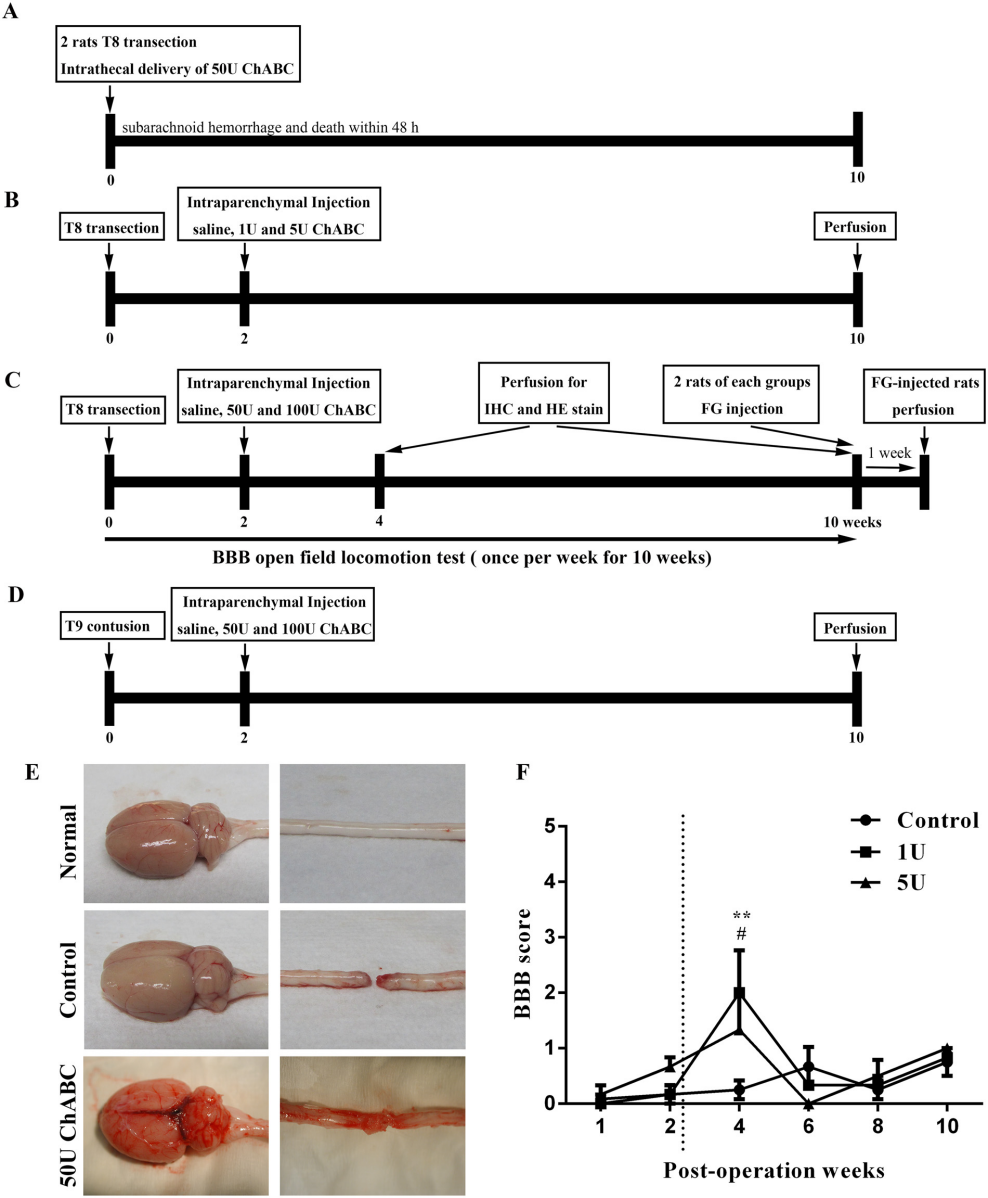
Experimental design of the chondroitinase ABC application. Flow chart of the lesion and treatment paradigm: intrathecal delivery of high-dose ChABC in the acute stage of complete spinal cord transection (A). Intraparenchymal injection of low-dose ChABC in the sub-acute stage of complete spinal cord transection (B). Intraparenchymal injection of high-dose ChABC in the sub-acute stage of complete spinal cord transection (C). Intraparenchymal injection of high-dose ChABC in the sub-acute stage of spinal cord contusion (D). Two rats that received high-dose ChABC (50 U) after spinal cord transection exhibited severe subarachnoid hemorrhage in the brain and spinal cord (not observed in normal or control rats), which resulted in the rats' death within 48 h (E). Hind limb locomotor function following intraparenchymal injection of low-dose ChABC in sub-acute SCI was evaluated by the BBB locomotor score. Statistical analysis demonstrated that locomotion was significantly improved at 4 weeks (1 U and 5 U) compared to the control group, but there was no significant functional recovery at any other time point (control group n = 6; 1 U and 5 U groups each n = 3; **p<0.01 for 1 U compared to the control; #*p*<0.05 for 5 U compared to the control, two-way ANOVA, Tukey post hoc test). The error bars denote the SEM.

In our previous study, the sustained delivery of low-dose ChABC via an intrathecal catheter (1 U/mL, injection volume: 6 μL for one animal) in acute-stage SCI rats partially improved their functional recovery according to the BBB scale, and subsequently, the levels of the CSPGs were significantly decreased. Furthermore, both anterograde neuronal tracing and immunohistochemistry data showed axon outgrowth across the injury site after the ChABC treatment [16]. We also investigated whether a high-dose ChABC treatment would promote functional recovery in the acute stage of SCI. Our data showed that high-dose ChABC treatment in the acute stage of SCI resulted in severe CNS subarachnoid hemorrhage (brain and spinal cord) (Fig 1E). In the clinic, the majority of SCI patients do not receive therapeutic treatment in the acute stage (i.e., the first 24 h after SCI). The strategies for treating sub-acute or chronic stages of SCI are more important, and the condition is totally different from that during the acute stage because of the dense glial scar that has formed at the lesion site. Because scar tissue is irregular in shape, it is difficult to surgically remove the glial scar from an injury site. In addition, removal of the glial scars may cause or enhance the process of secondary injury in the remaining tissue [9, 18]. Furthermore, axonal dystrophy and glial cell degeneration occur in the lesion site [8]. The scar tissue, which occludes the tip of the intrathecal delivery system, accumulates at the injury site in the sub-acute or chronic stages of SCI [19]. This process may be limited to the deep regions of the lesioned cord [20]. To avoid the limitation of ChABC diffusion, intraparenchymal injection was chosen to ensure that ChABC would be delivered directly into the tissue in the sub-acute stage. Initially, low-dose ChABC was applied in the sub-acute stage of SCI rats, but there was no significant functional recovery (Fig 1F). It is reasonable to hypothesize that the concentration of ChABC was too low to digest the densely formed glial scar in the sub-acute or chronic stages of SCI. Previous studies have reported on high-dose ChABC treatments in a chronic cervical hemisection rat model [21] with sub-acute or chronic thoracic cervical hemisection [22, 23]; these studies indicated that treatment with high-dose ChABC enhanced axonal regrowth and functional recovery. However, no studies have used high-dose ChABC treatments in the sub-acute complete transection model, which is a more severe SCI animal model. Therefore, in the current study, we examined the effects of high-dose ChABC treatments in the sub-acute stage of complete spinal cord transection in rats. We first investigated whether the administration of high-dose ChABC was safe for the SCI rats and did not result in subarachnoid hemorrhage within the CNS. Then, we investigated the effects of the high-dose ChABC treatment in the sub-acute stage in (1) glial scar digestion and (2) axonal regrowth and whether this was sufficient to promote functional recovery in the complete transection model.

## Materials and Methods

### Animals

A total of 58 young adult female Sprague-Dawley rats (200–250 g) were used in this study. Before surgery, the rats were housed in groups of 6 in a standard cage under standard controlled environmental conditions (22.5 ± 2°C, 60% relative humidity, 12 h light/dark cycle) and with free access to water and food. After surgery, all efforts were made to minimize the animals’ suffering, and 2 rats were housed per cage under the same conditions. All procedures involving animals were approved by the Animals Committee of Taipei Veterans General Hospital (Permit Numbers IACUC 2013–187 and IACUC 2014–002) and were in accordance with the Guide for the Care and Use of Laboratory Animals of the National Institutes of Health.

### Surgical procedures

The surgical procedures for complete spinal cord transection have been described previously [16]. The animals were anesthetized with inhaled isoflurane (5% for induction, 1–2% for maintenance) in ambient air (2 L/min for induction, 1 L/min for maintenance) using an anesthetic vaporizer (Vopamatic, A. M. Bickford Inc., New York, NY, USA), and their body temperature was maintained at 37°C during surgery with a homoeothermic blanket.

In the current study, 90% of rats presented with hematuria after transection, contusion, or ChABC intraparenchymal injection, which disappeared after 3 or 4 days. Two rats that received intrathecal delivery of high-dose ChABC in the acute stage of SCI died unexpectedly within 48 h, with severe CNS hemorrhage after the 50 U ChABC delivery. To clarify whether the hemorrhage and death were due to the acute stage delivery of high-dose ChABC, the CNS was harvested without perfusion from an additional normal rat and T8 complete transection rat after two days and compared to those of the high-dose ChABC-treated rats (Fig 1E).

After a skin incision, the rats received a T8 total laminectomy to expose the spinal cord. The spinal cord was completely transected with a microsurgical stab knife (Katena Products, Inc., USA), and both stumps were lifted to complete the spinal cord transection. After transection, the spinal cord was retracted from the lesion site and resulted in an approximately 2 mm gap. The wound was closed in layers. After surgery, the rats were placed on a homoeothermic blanket for the first week. The animals were monitored twice per day for infections, general health and mobility throughout the post-injury survival period. The bladders were manually expressed twice daily. Prophylactic antibiotics (0.5 mL/day, subcutaneous, Borgal 6.5%, Intervet, Whitby, Canada) were administered once daily for the first week, and 0.9% normal saline was subcutaneously administered once daily for 3 days post-surgery. The analgesic acetaminophen (65 mg/kg, Taiwan Veterans, Taiwan) was subcutaneously delivered once daily for 2 days post-surgery.

For spinal cord contusions, an incision was made in the skin and longitudinal incisions were made on the left and right side of the T8-T12 vertebrae spinous processes. The rats received a T9-T10 total laminectomy to expose the spinal cord without disrupting the dura. The spinous processes at T8 and T11/12 were fixed with clamps to prevent movement during the contusion. The exposed cord was then severely contused by dropping a 10.0 g rod on the exposed T9 vertebra from a height of 50 mm using the NYU-MASCIS (New York University-Multicenter Animal Spinal Cord Injury Study) weight-drop impactor [24]. The wound was closed in layers and the rats cared for as described above.

### Intrathecal delivery of chondroitinase ABC

The experimental procedures of the ChABC application are shown in Fig 1A. Two rats were used to evaluate the effect of the high-dose ChABC treatment by intrathecal delivery in the acute SCI stage as described previously [16]. Briefly, a 30 gauge catheter was inserted intrathecally through the opening of the foramen magnum up to the T8-T9 level; the external portion of the catheter was used to inject ChABC. One week after the catheter insertion, the rats received a T8 transection as described above. The catheter was cut at the T8 level to infuse ChABC. The wound was closed in layers. After surgery, ChABC was administered every other day for 2 weeks (a total of eight times, with 6 μL each time) under inhalation anesthesia.

### Intraparenchymal injection of chondroitinase ABC

The animals were administered ChABC two weeks (sub-acute stage) after the operation and following a second behavioral test. The transected rats were randomly divided into a 0.9% normal saline control group (n = 4 for 4 weeks; n = 7 for 10 weeks) or low-dose ChABC: (1) 1 U and (2) 5 U ChABC (Fig 1B, each n = 3 for 10 weeks) and high-dose ChABC: (1) 50 U and (2) 100 U ChABC (Fig 1C, each n = 4 for 4 weeks; n = 7 for 10 weeks) subgroups. The rats with a spinal cord contusion were randomly divided into three groups: (1) a 0.9% normal saline control group and (2) 50 U and (3) 100 U ChABC groups (Fig 1D, each n = 4 for 10 weeks). A second operation was performed to deliver either ChABC (C3367, Sigma-Aldrich, St. Louis, MO, USA) or, for the control group, normal saline by intraparenchymal injection. The rats were re-anesthetized, and the spinal cords were re-exposed at the thoracic vertebrae (7^th^ and 9^th^ for transection; 8^th^ and 10^th^ for contusion). ChABC or normal saline were injected at a 1 mm depth in the midline of the spinal cord (1 mm rostral and caudal to the lesion center for the transection model; 2 mm rostral and caudal to the lesion center for the contusion model, 5 μL per injection) using a 32 gauge microsyringe (Hamilton, USA, 1 μL/min) mounted on a stereotactic micromanipulator. The needle was left in the tissue for an additional 5 min to avoid leakage of the solution. The wounds were closed in layers and the rats cared for as described above.

### Behavioral assessments

After spinal cord injury, the animals underwent a behavioral test once per week for 10 weeks. The rats were observed in the open field for 5 min and recorded by videotape for later reference if necessary. Two independent examiners were blinded to each group and evaluated the animals’ behaviors. The hind limb locomotor behavior of the rats was evaluated by the Basso, Beattie and Bresnahan (BBB) open field locomotor test [25]. The open field locomotor activity score was determined by observation and scoring the behaviors involving the trunk, tail and hind limb. The scores ranged from 0 to 21 (0 = no movement; 21 = normal movement). The scores of the ChABC-treated and control rats at each time point were compared for statistical analysis using GraphPad Prism v6.01 software and two-way ANOVA with Tukey-Kramer post hoc tests.

### Retrograde Fluoro-Gold tracing

The retrograde tracer Fluoro-Gold (FG, Fluorochrome Inc., Denver, CO, USA) was used to confirm axonal regeneration after spinal cord transection, and the axonal tracer techniques were performed as previously described [26]. After the final behavioral test, three rats in each of the saline, 50 U and 100 U ChABC-treated groups were randomly selected for retrograde FG tracing. In addition, three normal rats were treated with FG to establish the basal level of FG labeling. Ten weeks after spinal cord transection, the 13^th^ thoracic spinal cord was exposed by laminectomy and completely transected with microscissors to create a lesion gap. The gelfoam was soaked in FG solution (4% in normal saline) and inserted into the gap, and petroleum jelly was then placed on the spinal cord and FG gelfoam to prevent FG diffusion. The rats survived one week for retrograde FG transport to the motor and sensory nuclei in the supraspinal region and cortex, and then the tissue was prepared as described below.

### Tissue preparation

At 2 and 8 weeks after treatment, the animals were deeply anesthetized with sodium pentobarbital (0.3 mL/100 g) and fixed by intracardiac perfusion using 4% paraformaldehyde in phosphate-buffered saline (PBS). The brains and spinal cords, including the rostral and caudal stumps (each 10 mm) separated by the transection gap (~0.5 mm), were removed and postfixed overnight in 4% paraformaldehyde at 4°C. The tissue was cryoprotected with 15% sucrose overnight, transferred to 30% sucrose for 2 days, immersed in embedding compound, and quickly frozen in a freezer at -80°C. Serial horizontal sections of the tissues were cut at 20 μm on a cryostat and mounted on poly-L-lysine-coated slides for immunostaining.

For FG tracing, the rats were perfused with 4% paraformaldehyde in 0.1 mM phosphate buffer (PB), after which the brains and spinal cords were removed and postfixed overnight in 4% paraformaldehyde in PB at 4°C. The tissue was cryoprotected with 15% sucrose in PB overnight, transferred to 30% sucrose in PB for 2 days, and then embedded in mounting medium. Serial transverse sections of the brains were cut at 20 μm, and the spinal cords were sectioned as described above.

### Immunohistochemistry and immunofluorescence

The sections were stained with hematoxylin and eosin to measure the length and area of the lesion site. To investigate the changes in the glial scar components, the sections were incubated with 2% bovine serum albumin in PBS for 1 h. The undigested CSPGs were visualized using the CS-56 monoclonal antibody (1:500; C8035, Sigma, St. Louis, MO, USA), whereas the digested CSPGs were visualized using the 2B6 antibody (1:500; 2704321, Seikagaku Corporation, Japan). The C4S antibody (1:200, MAB2030, CHEMICON International, Inc., USA) was used to label chondroitin 4-sulfate, and the C6S antibody (1:100, MAB2035, CHEMICON® International, Inc., USA) labeled chondroitin 6-sulfate overnight at 4°C. The appropriate secondary antibodies and the avidin-biotin-peroxidase complex (Vectastain Elite ABC kit; Vector Laboratories Inc., USA), with appropriate chromagens, were used to visualize the antibodies and examined by Leica confocal microscopy. To label the axons that regenerated across the injury site, double immunofluorescence labeling was performed using a β-III tubulin mouse monoclonal primary antibody (Tuj1, 1:500, MMS-435p; Covance, USA) to label the newly regrown axons [27]. The reactive astrocyte and glial scar were visualized using the glial fibrillary acidic protein rabbit polyclonal antibody (GFAP, 1:500, Z0334, Dako, Carpinteria, CA, USA) [28]. All sections were incubated with the primary antibody overnight at 4°C. The following day, the sections were incubated with an Alexa Fluor 488-conjugated donkey anti-rabbit (1:200, A21206, Molecular Probes, USA) or Cy3-conjugated donkey anti-mouse secondary antibody (1:200, 715-166-152, Jackson ImmunoResearch. Inc., PA, USA) and examined by fluorescence microscopy. To identify the FG-positive neurons, the brain and spinal cord sections were rinsed with 0.1 mM PB, blocked with 2% bovine serum albumin in 0.1 mM PB with 0.2% Triton X-100 at room temperature for 1 h, and incubated overnight with a rabbit anti-FG antibody (1:3000, Fluorochrome Inc., Denver, CO, USA) at 4°C. The next day, the sections were incubated with an anti-rabbit IgG secondary antibody (Vector Laboratories Inc., USA) and the signal was amplified with an ABC kit and visualized with the appropriate chromagens. The light microscopy images were taken using a ZEISS Axioskop 2 MOT microscope attached to a digital camera (Axiocam HRc, Zeiss) using the Zeiss Axiovision v4.7 software.

### Quantification of the glial scar components

To evaluate the glial scar components that were digested by ChABC, the intensities of the spinal cord areas immunolabeled for CS56, 2B6, C4S and C6S were quantified using two mid-sagittal sections spaced 5 mm caudal and rostral to the injury epicenter. The staining intensity thresholds for each antibody were determined after all of the images were acquired to optimize the signal-to-noise ratio for each antibody. The signal intensity threshold selected was 30 to 170 (within the full range of intensities extending from 0 to 255) for the immunoreactivity of CS56, 2B6, C4S and C6S. The areas with intensities within this threshold range were measured and normalized to the total tissue areas. Statistical analyses were performed using GraphPad Prism v6.01. One-way ANOVA and Tukey-Kramer post hoc tests were used.

### Quantification of the length and area of the lesion site

To determine whether the rostral-caudal extent of the lesion area changed after ChABC treatment, we used a mid-longitudinal section to compare the profile of the lesion length and area at 4 and 10 weeks after spinal cord transection. Hematoxylin and eosin staining defined the lesion area and length range. ImageJ “Straight” was used to evaluate the lesion length from the rostral to distal stump. Lesion areas containing cysts and cavities were randomly selected and analyzed using ImageJ software. As the areas of the entire longitudinal sections varied in the different treatment groups, the sum of the lesion areas was calculated and normalized by averaging the total number of longitudinal sections at 5 mm rostral and distal to the lesion midline. Statistical analyses were performed using GraphPad Prism v6.01. One-way ANOVA and Tukey-Kramer post hoc tests were used.

### Quantification of the axonal outgrowth density

The axonal outgrowth in each treatment group was quantified at 4 and 10 weeks post-operation by determining the density of the β-III tubulin-positive axons at the lesion sites in immunofluorescence images using ImageJ software. GFAP expression was used to identify the spinal cord stump and the glial scar around the border of the SCI. The GFAP- and β-III tubulin-positive images were captured on a ZEISS Axioplan 2 MOT epifluorescence microscope attached to a digital camera (Axiocam MRm, Zeiss) using Zeiss Axiovision v4.7 software. The digital images were exported to the ImageJ software (NIH Image, USA) to measure the level of the β-III tubulin signal between the rostral and caudal borders of the lesion area in two midline longitudinal sections (n = 4 per group). The selected signal intensity threshold was 25 for the β-III tubulin immunoreactivity (within the full range of intensities extending from 0 to 255). The β-III tubulin-positive areas with intensities higher than this threshold were calculated and divided by the total selected area (presented as a percentage of β-III tubulin). Statistical analyses were performed using GraphPad Prism v6.01. One-way ANOVA and Tukey-Kramer post hoc tests were used.

### Quantification of the retrogradely labeled neurons

To count the retrogradely labeled neuronal cells, two adjacent sections of the rostral mid-longitudinal spinal cord (10 mm) and coronal brain sections (from brain stem to the motor cortex) were examined using light microscopy as described above. Only neurons with visible punctate FG staining were counted, and the total number of FG-positive neurons in each target nuclei was counted for each rat. The mean number of FG-positive cells and standard error (SE) were calculated for the control versus the 50 U and 100 U groups. Statistical analyses were performed using GraphPad Prism v6.01. For the spinal cord, the variations among the normal, control and treatment groups were examined by one-way ANOVA with Tukey-Kramer post hoc testing. For the brainstem regions, the variations in treatment and distance in the normal, control and treatment groups were examined by two-way ANOVA with Tukey-Kramer post hoc testing. The numbers of FG-positive cells in the respective nuclei in all of the groups were summed and analyzed by linear regression.

## Results

### Intraparenchymal injection of high-dose ChABC in sub-acute SCI does not induce subarachnoid hemorrhage

We first tested intrathecal delivery of high-dose ChABC (50 U) in the acute stage of SCI in rats (n = 2). However, these two rats died within 48 h due to severe CNS hemorrhage after the delivery of 50 U ChABC. To clarify whether the hemorrhage and death were due to the acute-stage delivery of high-dose ChABC, the brains and spinal cords from an additional normal and T8 complete transected rat were harvested without perfusion after two days and compared to those of the high-dose ChABC-treated rats. Our results showed that severe subarachnoid hemorrhage in the brain and spinal cord was induced in the 50 U ChABC-treated rats (Fig 1E) but not in the normal or T8 transected rats. Because most SCI patients are treated in the sub-acute or chronic stages, when the dense glial scar has already formed, there is limited diffusion of the intrathecally delivered treatment. Thus, intraparenchymal injection of ChABC was chosen for use in the sub-acute stage of SCI. First, we evaluated the effects of a low-dose ChABC treatment in the sub-acute stage of SCI. Although some functional recovery occurred in the ChABC-treated rats at 2 weeks, there was no significant long-term functional recovery (Fig 1F, 6–10 weeks). Hence, we used high-dose ChABC in the sub-acute stage of SCI to confirm whether intraparenchymal delivery of high-dose ChABC would cause subarachnoid hemorrhage or death. In this study, all of the SCI rats survived after the 50 U and 100 U ChABC treatments in the sub-acute stage of SCI and did not display subarachnoid hemorrhage.

### Efficacy of sub-acute chondroitinase ABC treatment

**Fig 2:**
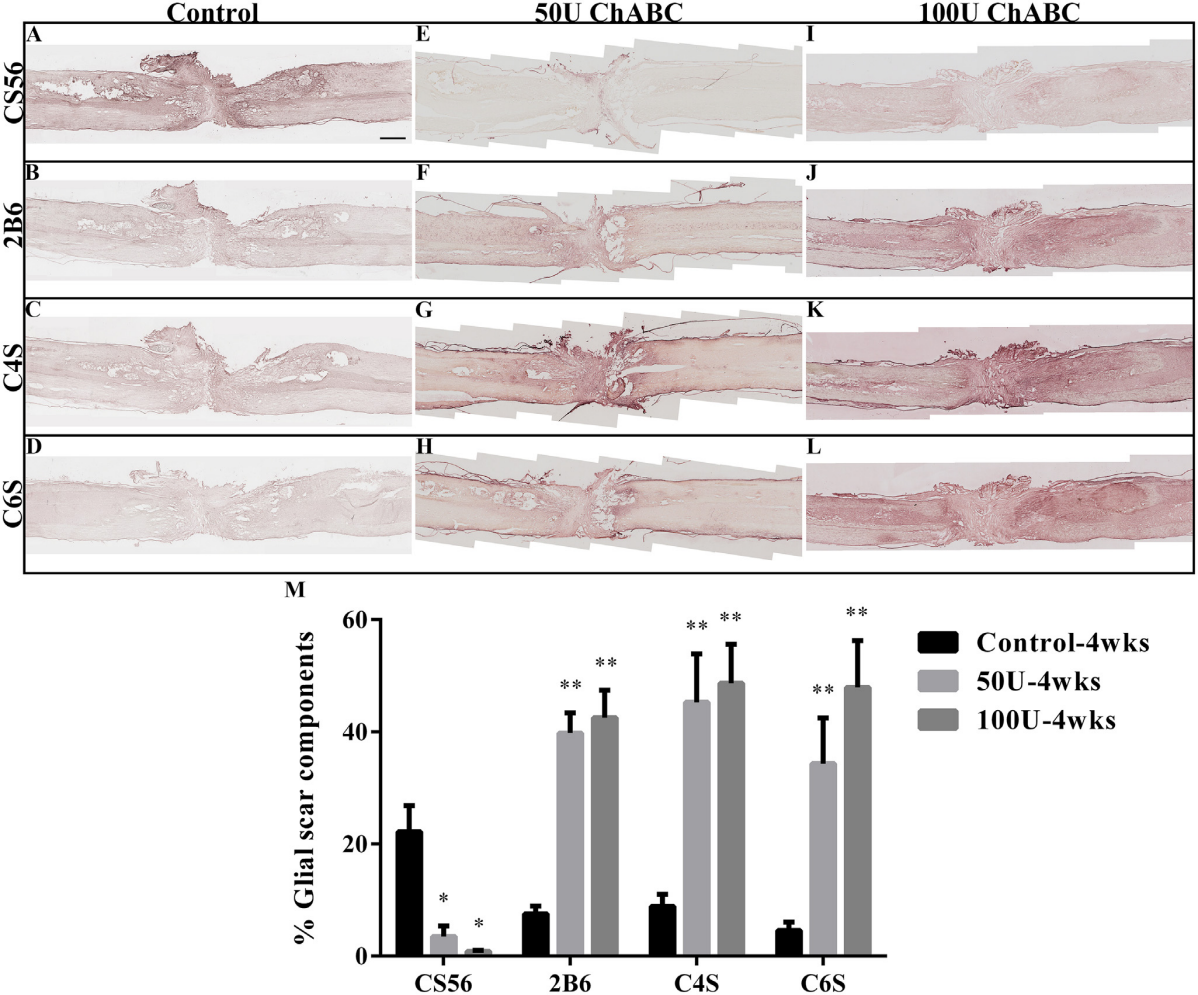
Expression of CSPGs in the high-dose ChABC treatment groups at 4 weeks after spinal cord transection. The figures present CSPG immunohistochemistry in longitudinal spinal cord sections at 4 weeks after parenchymal chondroitinase injections at 2 weeks after injury. The undigested CSPG, CS56 (A, E, I), and digested CSPGs, 2B6 (B, F, J), C4S (C, G, K), and C6S (D, H, L), were detected with specific antibodies. There was an abundance of CS56 immunoreactivity within the lesion site in the transected spinal cord (A). In contrast, the ChABC treatment dramatically decreased the CS56 immunoreactivity in the 50 U (E) and 100 U (I) groups. The digested CSPGs, which were detected by the anti-2B6, C4S and C6S antibodies, were significantly increased in the 50 U (F, G, H) and 100 U (J, K, L) groups compared to the control group (B, C, D). The histogram represents the quantitative analysis of the digested and undigested CSPG immunoreactivity at the injured site (M). There were significant differences in the levels of CS56, 2B6, C4S and C6S immunoreactivity between the ChABC-treated and control groups (each n = 4; *p<0.05; **p<0.01, one-way ANOVA, Tukey’s post hoc test), but there was no significant difference between the 50 U and 100 U ChABC-treated groups. The error bars denote the SEM. Scale bar = 1000 μm.

**Fig 3:**
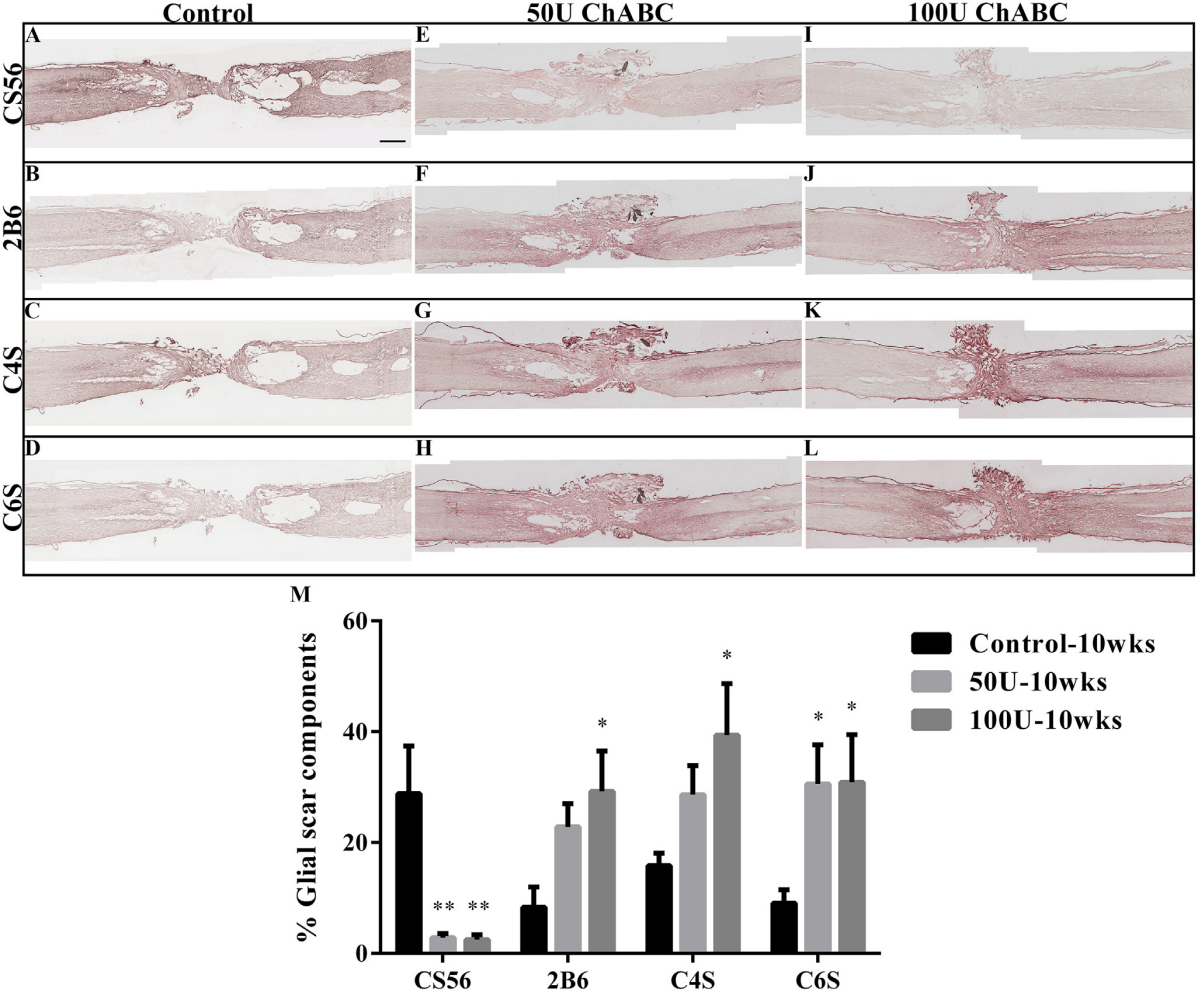
Expression of CSPGs in the high-dose ChABC treatment groups at 10 weeks after spinal cord transection. The figures present the CSPG immunohistochemistry in the longitudinal spinal cord sections at 10 weeks after parenchymal chondroitinase injections at 2 weeks after injury. The undigested CSPG, CS56 (A, E, I), and digested CSPGs, 2B6 (B, F, J), C4S (C, G, K), and C6S (D, H, L), were identified by specific antibodies. There was abundant CS56 immunoreactivity within the lesion site in the transected spinal cord (A). In contrast, ChABC treatment dramatically decreased the CS56 immunoreactivity in the 50 U (E) and 100 U (I) groups. The digested CSPGs, which were detected by the anti-2B6, C4S and C6S antibodies, were significantly increased in the 50 U (F, G, H) and 100 U (J, K, L) groups compared to the control group (B, C, D). The histogram represents the quantitative analysis of the digested and undigested CSPG immunoreactivity in the injured site (M). There were significant differences in the levels of CS56, 2B6, C4S and C6S immunoreactivity between the ChABC-treated and control groups (each n = 4; *p<0.05; **p<0.01, one-way ANOVA, Tukey’s post hoc test), but there was no significant difference between the 50 U and 100 U ChABC-treated groups. The error bars denote the SEM. Scale bar = 1000 μm.

CSPGs were expressed in the gliotic regions in and around the CNS injury site, and a large amount of CSPG was present two weeks after SCI [6]. Hence, we investigated whether the high-dose ChABC could effectively digest the CSPGs in the sub-acute stage of SCI. To analyze whether ChABC digested the CSPGs, we used the anti-2B6, CS4 and CS6 antibodies to recognize different epitopes on the CSPGs. The undigested CSPGs were recognized by the anti-CS56 antibody. After treatment with high-dose ChABC for 2 weeks, the levels of CS56 were significantly decreased in the 50 U and 100 U ChABC-treated groups (3.49 ± 1.87 and 0.845 ± 0.19%, respectively) compared to the control group (22.16 ± 4.66%, *p*<0.01). The levels of digested CSPGs, as recognized by the 2B6 (50 U: 39.76 ± 3.59%; 100 U: 42.55 ± 4.88%; control: 7.55 ± 1.36%, **p<0.01), C4S (50 U: 45.30 ± 8.55%; 100 U: 48.67 ±6.94%; control: 8.86 ± 2.17%, **p<0.01) and C6S antibodies (50 U: 34.30 ± 8.17%; 100 U: 47.94 ± 8.33%; control: 4.57 ± 1.52%, **p<0.01) were significantly higher in the high-dose ChABC-treated groups than the control group at 4 weeks post-operation (Fig 2). However, the CS56, 2B6, C4S and C6S levels were not significantly different between the 50 U and 100 U ChABC-treated groups. Furthermore, the C4S immunoreactivity was mainly located at the injury site (Fig 2C, 2G and 2K), but the C6S immunoreactivity was primarily in the penumbra of the lesion site and spinal cord stump (Fig 2D, 2H and 2L). Eight weeks after the ChABC treatment, the levels of CS56 were still reduced in the high-dose ChABC-treated group compared to the control group (50 U: 2.85 ± 0.75%; 100 U: 2.48 ± 0.89%; control: 28.82 ± 8.58%, **p<0.01, Fig 3A, 3E and 3I). The expression of the digested CSPGs using the 2B6 (50 U: 22.81 ± 4.20%; 100 U: 29.20 ± 7.30%; control: 8.33 ± 3.65%, *p<0.05, Fig 3B, 3F and 3J), C4S (50 U: 28.62 ± 5.24%; 100 U: 39.38 ± 9.29%; control: 15.82 ± 2.25%, *p<0.05, Fig 3C, 3G and 3K), and C6S antibodies (50 U: 30.56 ± 7.05%; 100 U: 30.88 ± 8.58%; control: 9.06 ± 2.44%, *p<0.05, Fig 3D, 3H and 3L) were also higher in the high-dose ChABC-treated group than the control group. However, there were also no significant differences in CS4 and CS6 staining between the 50 U and 100 U ChABC-treated groups (Fig 3M). In the control group, little or no CS4 immunoreactivity was detected in the injury site at 10 weeks after injury (Fig 3C). These results suggest that the delivery of high-dose ChABC in the sub-acute stage effectively digested the CSPGs and altered the glial scar components after spinal cord injury.

### Chondroitinase ABC treatment prevented the shrinking of spinal cord stumps and decreased the extent and area of the lesion as well as cyst formation

**Fig 4:**
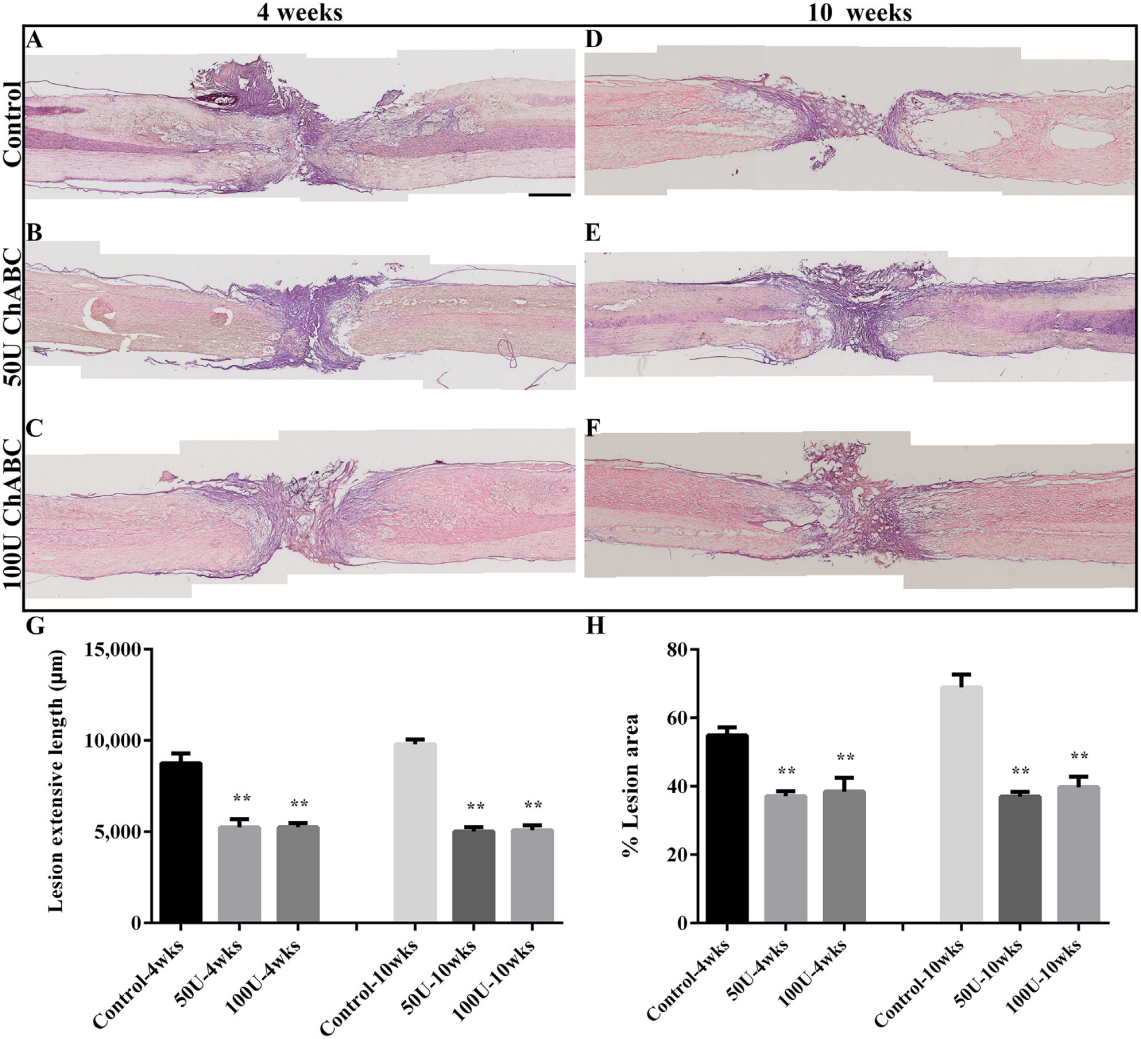
Photographs showing the cavities and cyst formation after T8 spinal cord transection. Longitudinal spinal cord sections from the control (A, D), 50 U (B, E) and 100 U (C, F) ChABC-treated groups were stained with hematoxylin and eosin (H&E) at 4 (A-C) and 10 (D-F) weeks after spinal cord injury. In the control group, many cavities and cysts (holes filled with hematoxylin-positive cells) had obviously appeared in the rostral and distal stump near the lesion site at 4 weeks after injury (A). Subsequently, the shrinking spinal cord tissue created additional cavities and cysts around the lesion site that became larger and farther from the spinal stumps at 10 weeks after injury (D). In ChABC-treated groups, cysts with hematoxylin-positive cells were also exhibited around the epicenter of the lesion site after treatment with 50 U (B) or 100 U ChABC (C), but fewer cavities had formed and extended at 4 weeks after spinal cord injury. In the 50 U (E) and 100 U (F) ChABC-treated groups, cysts also formed around the lesion site and extended a shorter distance from the spinal stumps compared to the control group at 10 weeks after SCI. The histograms represent measurements of the cavities' length between the rostral and caudal edges of the cavities (G) and the lesion area (H) in the spinal cord. The histograms show that treatment with 50 U and 100 U ChABC significantly decreased the length of the cavities compared to the control group at 4 and 10 weeks after spinal cord injury, but there was no significant difference between the ChABC-treated groups (n = 4 per group; **p<0.01, one-way ANOVA, Tukey’s post hoc test). The error bars denote the SEM.

After spinal cord injury, a glial scar and cystic cavities formed in the lesion sites and extended into the intact stumps. Previous studies have demonstrated that ChABC treatment reduces glial scar and cyst formation after spinal cord injury [29]. We used hematoxylin and eosin (H&E) staining to investigate the extent of the lesion and cyst formation. After spinal cord transection, the lesion length was dramatically decreased in the 50 U and 100 U ChABC-treated groups compared to the control group at 4 weeks (50 U: 5.23 ± 0.45 mm; 100 U: 5.24 ± 0.23 mm; control: 8.76 ± 0.53 mm, **p<0.01, Fig 4A, 4B, 4C and 4G) and 10 weeks (50 U: 5.01 ± 0.48 mm; 100 U: 5.08 ± 0.52 mm; control: 9.78 ± 0.56 mm, **p<0.01, Fig 4D, 4E, 4F and 4G) post-operation. The lesion area and cyst formation were also significantly decreased in the 50 U and 100 U ChABC-treated groups compared to the control group at 4 weeks (50 U: 37.12 ± 2.78%; 100 U: 38.43 ± 8.21%; control: 54.88 ± 4.66%, **p<0.01, Fig 4A, 4B, 4C and 4H) and 10 weeks (50 U: 36.94 ± 2.83%; 100 U: 39.7 ± 6.10%; control: 68.88 ± 7.58%, **p<0.01, Fig 4D, 4E, 4F and 4H) after spinal cord injury. However, there was no significant difference in the extent and area of the lesions between the 50 U and 100 U ChABC-treated groups at 4 and 10 weeks, respectively. Many hematoxylin-positive cells filled the cavities in the control group, suggesting that these cells may be involved in the process of secondary injury (Fig 4A). In the ChABC-treated groups, cysts were still present in the lesion site, but their size and length were significantly decreased compared to the control group. Moreover, there were fewer hematoxylin-positive cells in the lesion cavities after the ChABC treatment (Fig 4B and 4C) at 4 weeks post-operation. At 10 weeks after transection, there were large cavities in the spinal cord stumps and the tissue became atrophied at the lesion site in the control group (Fig 4D). In contrast, the cavities decreased and spinal tissue was more preserved in the ChABC-treated groups compared to the control group at 10 weeks post-operation (Fig 4E and 4F). These results imply that the ChABC treatment may have effectively reduced the secondary damage by decreasing the extent and area of the lesions, decreasing cyst formation, and preserving the spinal cord tissue after spinal cord injury.

### Treatment with high-dose chondroitinase ABC in the sub-acute stage promoted axonal outgrowth across the lesion site

**Fig 5:**
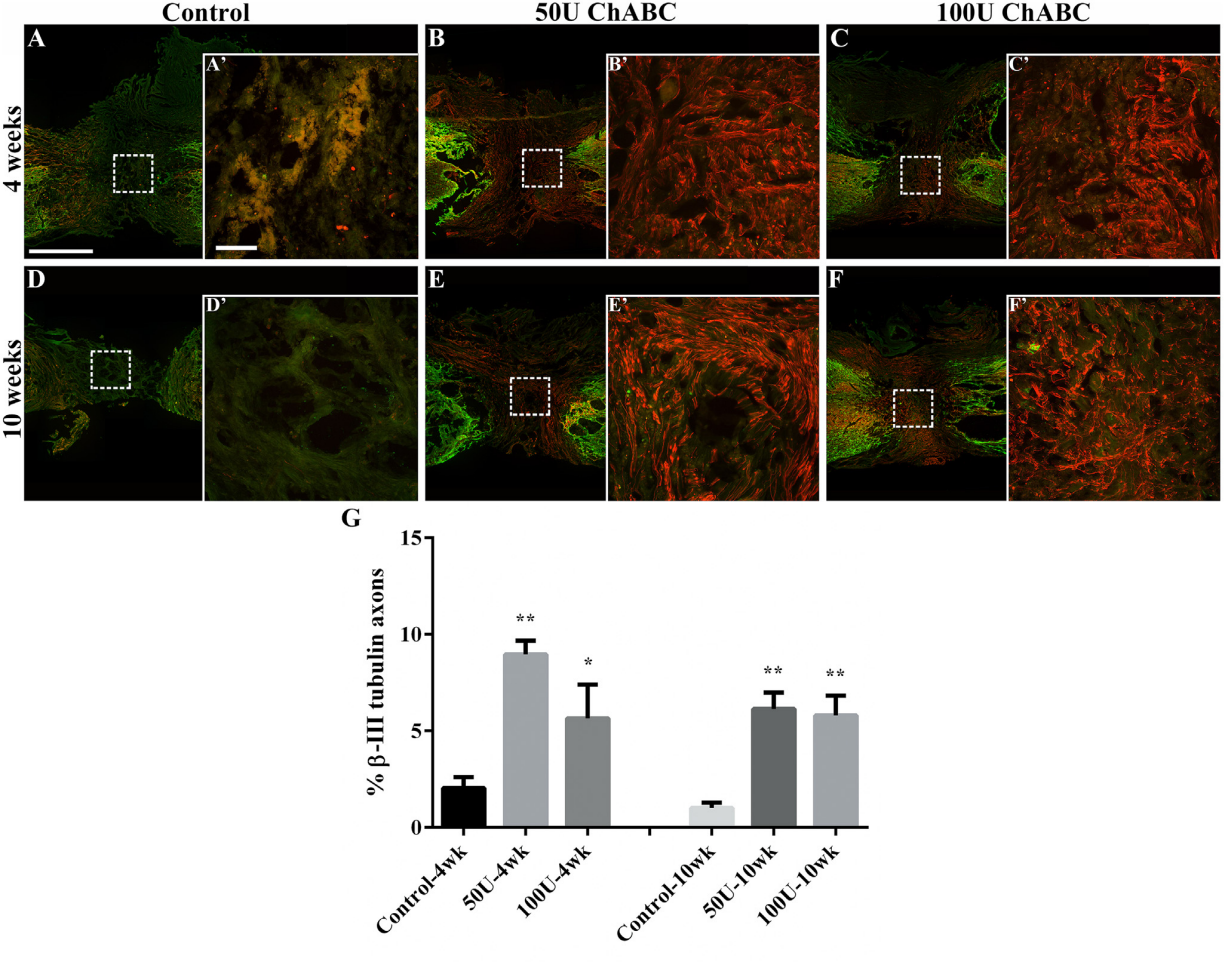
Axonal outgrowth in the injured spinal cord after high-dose ChABC treatment. Longitudinal spinal cord sections from the control (A, D) and the 50 U (B, E) and 100 U (C, F) ChABC-treated groups were labeled with β-III tubulin (red) and GFAP (green, astrocytes) at 4 (A-C) and 10 (D-F) weeks after spinal cord injury; the dotted square indicates the higher magnification image of the lesion epicenter in each group (A’-F’). The GFAP+ astrocytes were considered as the boundary of the glial scar in the lesion site. In the control group, some β-III tubulin axons were able to outgrow to the interface of the lesion site, and a few axons extended to the injury epicenter at 4 weeks after injury (A). The number of β-III tubulin-positive axons decreased and there was no detectable axon outgrowth in the lesion epicenter at 10 weeks after SCI (D). It is notable that the 50 U (B) and 100 U (C) ChABC-treated groups showed a greater number of outgrown axons that crossed the astrocyte-rich territory and filled in the lesion site at 4 weeks after SCI. At 10 weeks, there were still many β-III tubulin-positive axons in the proximal and distal stumps in the 100 U (F) ChABC-treated group, but they were decreased in the 50 U (E) ChABC-treated group. However, β-III tubulin-positive axon outgrowth was also observed in the lesion site in both the 50 U and 100 U ChABC-treated groups (E, F) at 10 weeks after injury. The histograms show the percentage of β-III tubulin-positive axons in the lesion site at 4 and 10 weeks after injury (G). Statistical analysis demonstrated that the levels of β-III tubulin were significantly increased in the 50 U and 100 U ChABC-treated groups compared to the control group at 4 weeks and 10 weeks after spinal cord injury (n = 4 per group; *p<0.05, **p<0.01, one-way ANOVA, Tukey’s post hoc test). The error bars denote the SEM. Scale bars: A-F = 1000 μm; A’-F’ = 100 μm.

Previous studies have demonstrated that ChABC treatment induced axonal outgrowth across the injury site [15, 16, 22]. Therefore, we investigated whether the high-dose ChABC treatment in the sub-acute stage had any effects on axonal outgrowth after spinal cord transection. The β-III tubulin antibody was used to recognize the outgrowth axons. Two weeks after the ChABC treatment, the number of β-III tubulin-positive axons was significantly increased in the injury site in the ChABC-treated groups compared to the control group (50 U: 8.95 ± 0.72%; 100 U: 5.66 ± 1.75%; control: 2.04 ± 0.56%, Fig 5A–5C’ and 5G). Although some β-III tubulin-positive axons were detected in the control group, these axons did not cross the injury site (Fig 5A and 5A’). At 8 weeks after ChABC treatment, β-III tubulin-positive axonal profiles were still detectable throughout the lesion scar in the ChABC-treated groups but not in the control group (50 U: 6.13 ± 0.85%; 100 U: 5.79 ± 1.02%; control: 1.00 ± 0.28%, **p<0.01, Fig 5D–5F’ and 5G). These results suggest that the ChABC treatment increased the permeability of the glial scar and prolonged axonal outgrowth.

### Treatment with high-dose chondroitinase ABC in the sub-acute stage promoted axonal regeneration to the function-related nuclei in the brain stem

**Fig 6:**
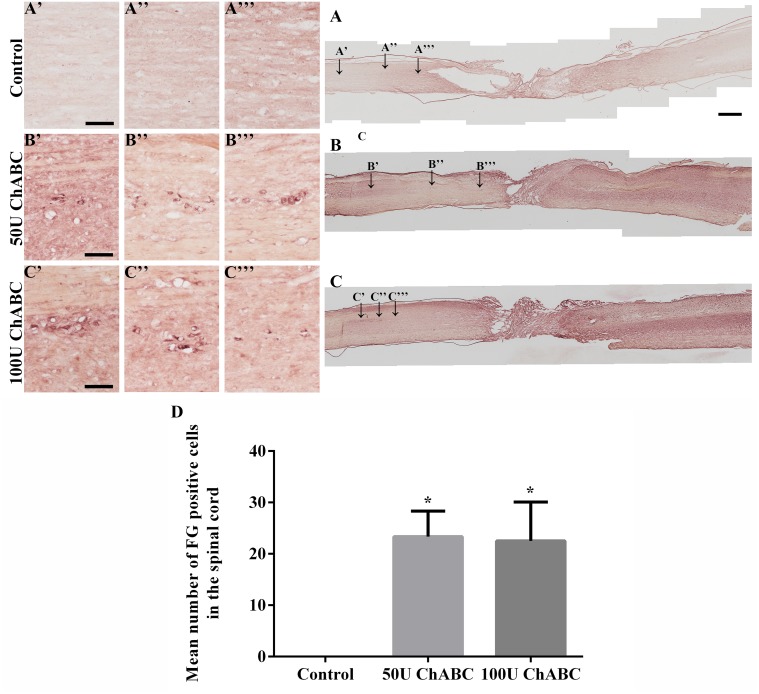
Fluoro-Gold (FG) retrogradely labeled neurons in the spinal cord. The presence of FG-positive neurons in the rostral stumps suggested that FG, which was injected at the T13 level, was absorbed by the anterogradely regenerating axons crossing the transection gap. In the control group, there were virtually no FG-positive neurons beyond the rostral stumps, demonstrating an absence of axonal regeneration (A, magnifications in A’-A”’). In contrast, there were many FG-positive neurons in the 50 U (B, magnifications in B’-B”’) and 100 U (C, magnifications in C’-C”’) ChABC-treated groups, showing that the ChABC treatment could promote axons to regenerate and cross the transection gap. Mean number of FG-positive neurons in the rostral spinal cord stump (D). There were no labeled neurons in the rostral spinal cord from the control groups. (n = 3 per group; *p<0.05, one-way ANOVA, Tukey’s post hoc test). The error bars denote the SEM. Scale bars: A, B, C = 1000 μm; A’-A”‘, B’-B”’, C’-C”’ = 100 μm.

Although the ChABC treatment promoted axon outgrowth across the lesion site, it is still unclear whether these outgrown axons reconnect to the function-related nuclei in the brain stem. The retrograde axonal tracer FG was used to clarify the axonal regeneration and reconnection. Three animals in each of the 50 U, 100 U and control groups were used for retrograde tracing at ten weeks after transection and after the final behavioral test was completed; three normal animals were used to determine the basal level of FG labeling. Gelfoam soaked with 4% FG solution was applied to the 13^th^ thoracic spinal cord, which was caudal to the lesion site. The signals of the FG-positive neurons were amplified and visualized by immunohistochemistry staining. FG-positive cells were detected in the rostral stumps in the ChABC-treated groups (Fig 6B, 6B’–6B’’’, 6C and 6C’–6C’’’). There was no significant difference in the number of FG-positive neurons (Fig 6D) between the 50 U and 100 U ChABC-treated groups (23.3 ± 5.0 and 22.5 ± 7.6, respectively). In contrast, FG-positive neurons were not observed in the rostral spinal cord stump (Fig 6A, 6A’–6A”’ and 6D).

**Fig 7:**
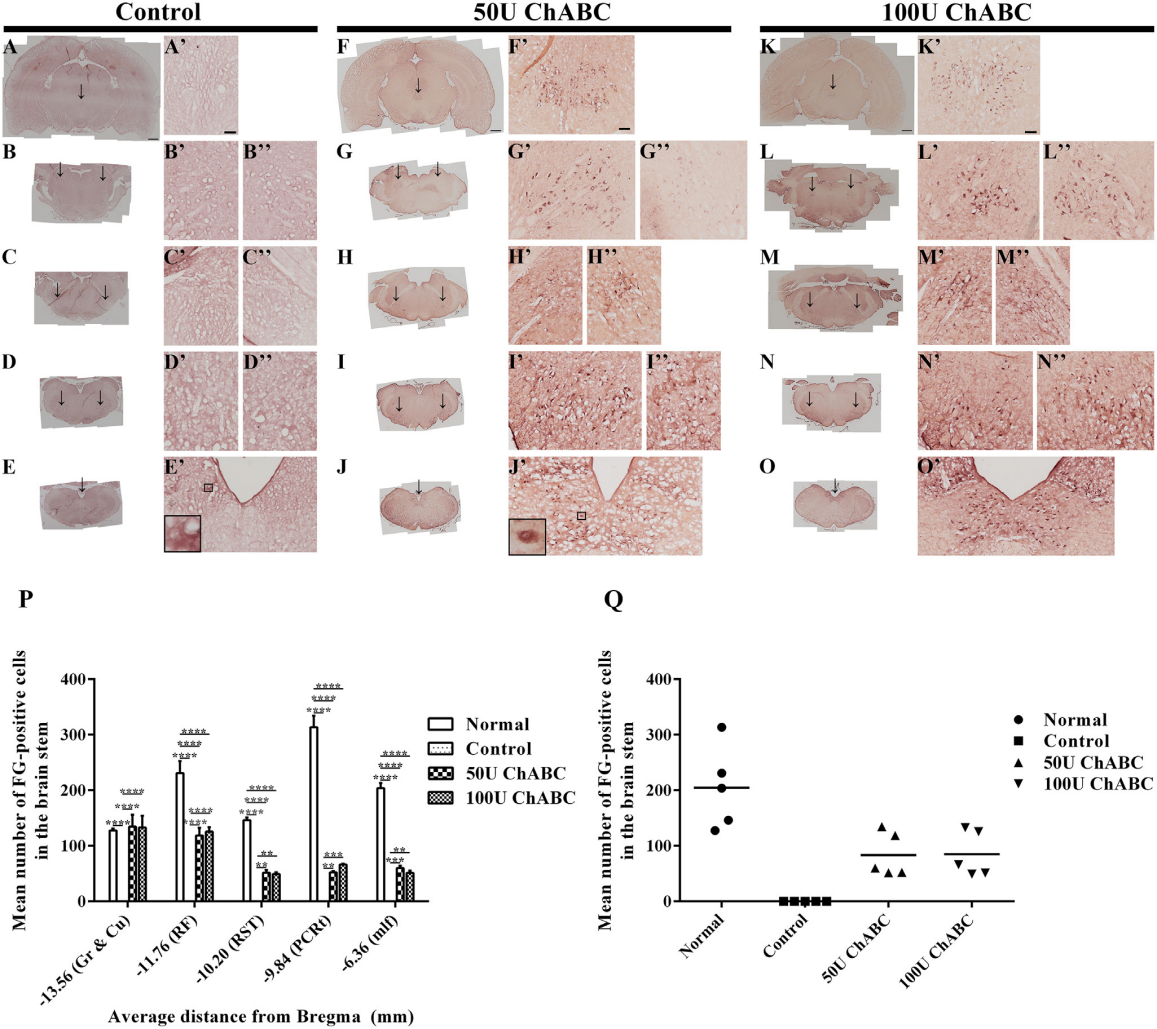
Fluoro-Gold (FG) retrogradely labeled neurons in specific brain stem nuclei. The coronal brain sections demonstrated that no FG-positive cells were detected in the brainstems in the control group (Fig A-E, magnifications in A’-E’, P). In the high-dose ChABC-treated groups, FG-positive neurons were detected in the descending/motor related pathway in the medial longitudinal fasciculus (50 U: F, magnifications in F’; 100 U: K, magnifications in K’) and rubrospinal tract (olivary body, 50 U: H, magnifications in H’, H”; 100 U: M, magnifications in M’, M”), and in the ascending/sensory related pathway in the parvicellular reticular nucleus (50 U: G, G’, G”; 100 U: L, magnifications in L’, L”), reticular formation (50 U: I, magnifications in I’, I”; 100 U: N, magnifications in N’, N”), and cuneate and gracile nuclei (50 U: J, magnifications in J’; 100 U: O, magnifications in O’). Mean number of FG-positive neurons in the target regions of the brainstem (P) (n = 3 per group; *p<0.05, **p<0.01, ****p<0.0001, two-way ANOVA, Tukey’s post hoc test). Linear regression analysis comparing the total number of FG-positive cells in the brainstem of the different treatment groups (Q). These reconnections of the descending and ascending pathways suggest that high-dose ChABC may partially promote functional recovery after spinal cord transection. Scale bars: A-J = 1000 μm; A’-J’ = 100 μm. mlf, medial longitudinal fasciculus; RST, rubrospinal tract; PCRt, parvicellular reticular nucleus; RF, reticular formation; Cu & Gr, cuneate and gracile nuclei, respectively. The boxed areas show the higher magnification images of a neuron with visible punctate FG staining (J’) and an artifact (E’).

In normal animals, the FG-positive cells were clearly detected within the brainstem nuclei, including the descending/motor-related pathway, including the red nucleus, medial longitudinal fasciculus (mlf), and rubrospinal tract (RST), and the ascending/sensory-related pathway, including the parvicellular reticular nucleus (PCRt), reticular formation (RF), and the cuneate and gracile nuclei (S1 Fig). However, FG-positive neurons were not detected in the control animals (transection-only, Fig 7A–7E, 7A’–7E’, 7P and 7Q). In contrast, FG-positive cells were detected in the descending/motor-related pathway in the longitudinal fasciculus (mlf; 50 U: Fig 7F and 7F’; 100 U: Fig 7K and 7K’) and rubrospinal tract (RST, olivary body, 50 U: Fig 7H, 7H’ and 7H”; 100 U: Fig 7M, 7M’ and 7M”) and in the ascending/sensory-related pathway in the parvicellular reticular nucleus (PCRt; 50 U: Fig 7G, 7G’ and 7G”; 100 U: Fig 7L, 7L’ and 7L”), reticular formation (RF, 50 U: Fig 7I, 7I’ and 7I”; 100 U: Fig 7N, 7N’ and 7N”), and cuneate and gracile nuclei (50 U: Fig 7J and 7J’; 100 U: Fig 7O and 7O’) after ChABC treatment. However, there were still no FG-positive cells in the red nucleus and motor cortex in the ChABC-treated groups.

The number of FG-positive neurons in the medial longitudinal fasciculus (normal: 203.8 ± 9.3; control: 0; 50 U: 58.3 ± 13.0; 100 U: 51.2 ± 13.3), rubrospinal tract (normal: 146.3 ± 4.4; control: 0; 50 U: 51.5 ± 12.4; 100 U: 49.33 ± 11.6), parvicellular reticular nucleus (normal: 313.3 ± 20.8; control: 0; 50 U: 52.17 ± 12.5; 100 U: 66.0 ± 14.0), reticular formation (normal: 230.7 ± 21.9; control: 0; 50 U: 118.3 ± 26.9; 100 U: 125.5 ± 27.0) and cuneate and gracile nuclei (normal: 127.7 ± 3.7.3; control: 0; 50 U: 134.5 ± 14.12; 100 U: 132.8 ± 14.0) was significantly higher in the normal group than the other groups. Two-way ANOVA analysis demonstrated that the major variation factor is the ChABC treatment rather than the distance between the nuclei and the transection site (Fig 7P). There was no significant difference between the 50 U and 100 U ChABC-treated groups (Fig 7P). Linear regression analysis revealed that the basal (normal) number of FG-positive cells was significantly decreased to zero after spinal cord transection (control) (Fig 7Q, normal: 204.4 ± 33.0; control: 0). The total number of FG-positive cells was dramatically increased in the ChABC-treated groups compared to the control group (50 U: 83.27 ± 17.9; 100 U: 84.97 ± 18.31). These results suggested that the high-dose ChABC treatment promoted axonal regeneration across the injury site and the axons reconnected to the function-related nuclei in the brainstem. In contrast, the rubrospinal and corticospinal neurons are more refractory than brainstem neurons, which could not regenerate and cross the transection gap.

### High-dose chondroitinase ABC treatment improves functional recovery

**Fig 8:**
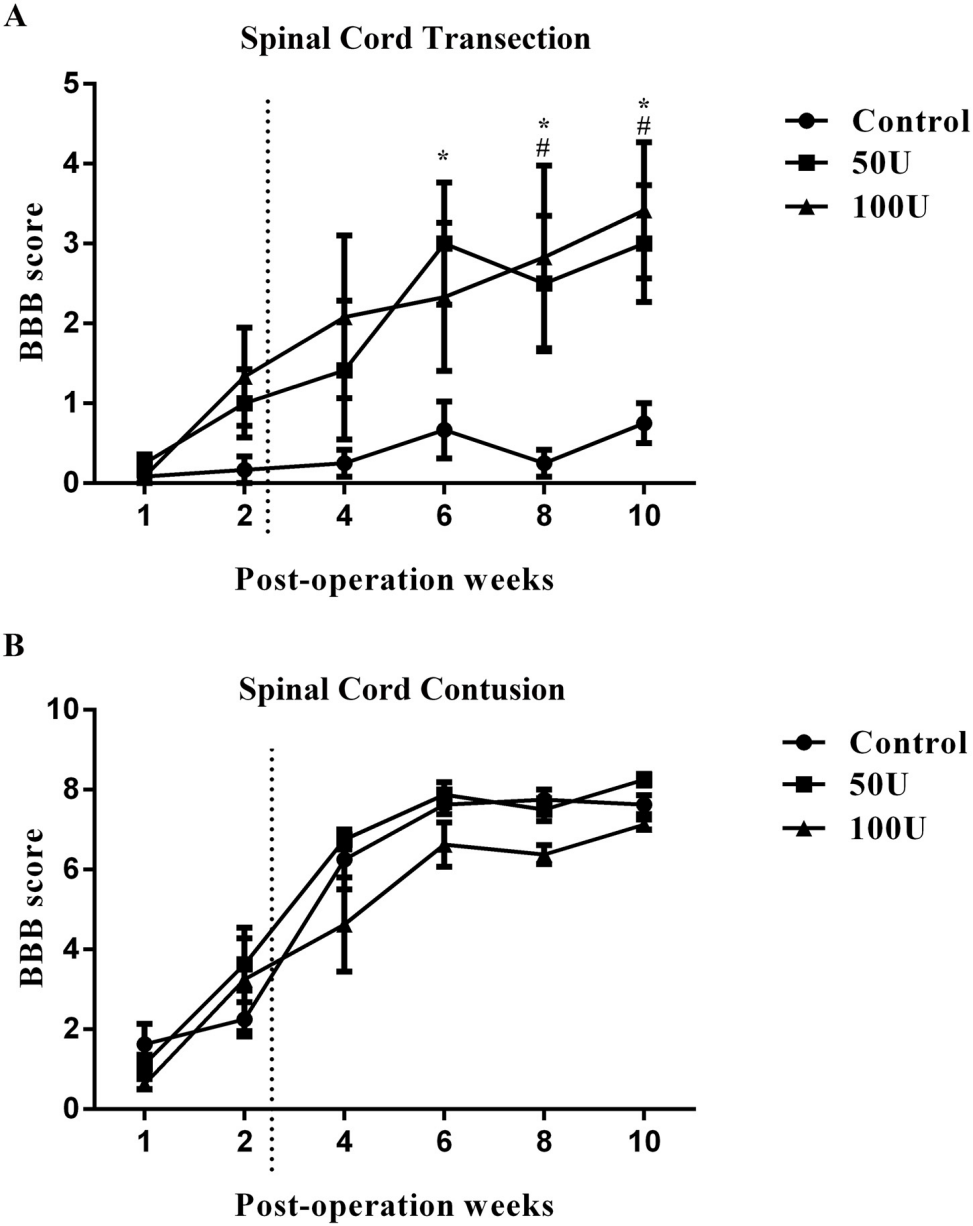
Time course of functional recovery in the high-dose ChABC-treated and control groups. The dotted line indicates the intraparenchymal injection of high-dose ChABC at 2 weeks after spinal cord transection. The BBB score shows that there was no significant improvement of the BBB score in the control group. In contrast, locomotion mildly improved with the 50 U and 100 U ChABC treatments. Statistical analysis demonstrated that locomotion significantly improved at 6 (50 U), 8 and 10 weeks (50 U and 100 U) compared to the control group, but there was no significant difference between the 50 U and 100 U ChABC-treated groups (A). After spinal cord contusive injury, there was no significant functional recovery in the ChABC-treated groups compared to the control group (B) (for transection, n = 6 per group; for contusion, n = 4 per group, *p<0.05 for 50 U compared to the control; #*p*<0.05 for 100 U compared to the control, two-way ANOVA, Tukey’s post hoc test). The error bars denote the SEM.

Next, we evaluated the effects of high-dose ChABC treatment on functional recovery using the BBB open field locomotor score [25]. Initially, after spinal cord transection, the hind limbs of the SCI animals were completely paralyzed. There was no significant difference between the control group and the ChABC-treated groups in the first four weeks. However, slight recovery, represented by spontaneous movements of three joints of the hind legs, was found over the next two weeks after ChABC treatment. Behavioral testing was performed until 10 weeks following the lesion (Fig 8A). At 6 weeks after spinal cord transection, the 50 U (3.0 ± 0.8) ChABC-treated group displayed significant functional improvement compared to the control group (0.6 ± 0.4). The improvement in locomotor function lasted throughout the experimental period in the ChABC-treated groups at 8 and 10 weeks (8 weeks, 50 U: 2.5 ± 0.8, 100 U: 2.8 ± 1.1; 10 weeks, 50 U: 3.0 ± 0.7, 100 U: 3.4 ± 0.9) compared to the control group (8 weeks: 0.25 ± 0.2; 10 weeks: 0.75 ± 0.2). The current results suggest that treatment with high-dose ChABC in the sub-acute stage at least partially improved the locomotor function after spinal cord transection. Furthermore, we used the spinal cord contusion injury model to investigate whether high-dose ChABC treatment could improve functional recovery. In both ChABC-treated groups, the locomotor recovery reached a peak and achieved average scores of 7 to 8 at 10 weeks after the contusion injury. However, there were no significant difference between the control and ChABC-treated groups throughout the experimental period (Fig 8B). These results indicated that functional recovery was not enhanced by the high-dose ChABC treatment after spinal cord contusion injury.

## Discussion

In the present study, we first examined the effects of high-dose ChABC treatment in the sub-acute stage in rats with a complete spinal cord transection. Our data showed that the intraparenchymal high-dose ChABC treatment significantly digested the CSPGs, decreased the extent and area of the lesion and decreased cyst formation. These protective effects preserved the spinal cord tissue, enhanced axon outgrowth throughout the injury site, and significantly improved functional recovery in the complete spinal cord transection model.

### Designing the treatment protocol and animal models

Therapy for SCI in the sub-acute and chronic stages faces serious challenges, such as the accumulation of glial scar tissue, cyst formation, regression of the injured neurons [30–32], and a shift in the microenvironment to inhibit axonal regeneration at the lesion site. Our previous study showed that sustained administration of low-dose ChABC in the acute stage of spinal cord injury could promote axonal regeneration and partially improve functional recovery [16]. Furthermore, we investigated whether high-dose ChABC treatment would increase the efficiency of glial scar digestion. However, our data showed that intrathecal delivery of high-dose ChABC in the acute stage of SCI resulted in severe CNS subarachnoid hemorrhage and death within 48 h (Fig 1E). To clarify whether T8 transection would also induce CNS subarachnoid hemorrhaging, the brains and spinal cords were harvested from the normal and T8-transected rats at 2 days after surgery and compared to the acute 50 U ChABC-treated rats. Our results verified that the T8 transection operation did not induce CNS subarachnoid hemorrhage. We suggest that complete spinal cord transection is a severe model resulting in the breakdown of the blood brain barrier, cerebrospinal fluid (CSF) leakage, and bleeding at the lesion site. Consequently, with the intrathecal delivery of high-dose ChABC immediately after SCI, the agent may diffuse into the leaked CSF and bleed into the intact spinal cord and brain. A previous report demonstrated that ChABC not only digested chondroitin sulfate but also the dermatan sulfate found in the blood vessels of the brain surface and meninges [33]. In addition, ChABC disrupted the blood vessel basement membrane in the acute stage after CNS injury [34]. Therefore, when the high-dose ChABC diffused into the brain with the CSF and blood, it may have caused damage to the blood vessels of the brain and resulted in subarachnoid hemorrhage. In the sub-acute and chronic stages of spinal cord injury, a dense, irregular glial scar formed and infiltrated the surrounding tissue in the lesion site, which was difficult to remove by surgery. Therefore, we assumed that parenchymal administration of ChABC into the rostral and caudal areas of the injury site may sufficiently digest the glial scar and promote axonal regrowth and functional recovery. At first, we used low-dose ChABC as explained in our previous report [16]. Although there was some functional recovery after 2 weeks of ChABC treatment, there was no long-term functional recovery (6–10 weeks). We concluded that the low-dose ChABC treatment could digest part of the glial scar and promote some axonal outgrowth in the early stage. However, the enzymatic activity was decreased by the animals’ body temperature [35, 36], and the newly synthesized glycan that re-accumulated with the remaining CSPGs may continuously obstruct axonal outgrowth. Several studies have used a high dose of ChABC to evaluate the effects in the sub-acute and chronic stages of SCI [21–23]. Therefore, we further investigated whether treatment with 50 U or 100 U of ChABC in the sub-acute stage of SCI would more effectively promote axonal outgrowth and functional recovery. However, our results showed that there was no significant difference between the 50 U and 100 U doses of ChABC. We suggested that the major CSPGs of the glial scar, such as neurocan, brevican and versican, peaked at 2 weeks after SCI [6] and that the glial scar had matured in the lesion site [34]. According to the current results, 50 U ChABC was considered to be sufficient to digest most of the CSPGs in the lesion site in sub-acute SCI, and similar results were also obtained in the 100 U ChABC group (Figs 2 and 3).

A number of *in vivo* studies have assessed the effects of ChABC treatment in rodent models with a partial spinal cord transection [15, 21, 22, 37, 38]. These studies demonstrated that ChABC promotes axonal regeneration and functional recovery after spinal cord injury. Although spinal hemisection has been used in animal models to study the effects of ChABC, there is large variability among different surgeons, and, therefore, it has been difficult to distinguish between the regenerating axons and the sprouting spared axons [39]. The contusion model, which is more clinically applicable, has also been used to study the effects of ChABC treatment [17, 22, 40]. However, the animals did not exhibit significant functional recovery following ChABC treatment in this contusion model [17]. Our studies showed similar results, as there was no significant functional recovery in the ChABC-treated groups compared to the control groups after contusive spinal cord injury (Fig 8B). Although most studies have suggested that a high-dose ChABC treatment could not promote axonal regrowth in the contusion model [22], other studies have shown motor and bladder function improvement after ChABC treatment [41]. Moreover, some studies have indicated that ChABC gene delivery via intraspinal injection in the contusion model promotes the digestion of CSPGs, modulates the secondary injury processes, and improves functional recovery [42]. The pathological processes of the contusion model are complex and cause severe tissue damage and gliosis that expand from the injury epicenter. In the contusion model, it may be necessary to prolong the infusion period of ChABC or combine ChABC with other therapeutic strategies [22, 43]. It is also difficult to distinguish the regenerating axons from the spared axons at the spinal cord injury site [39]. The complete transection model is the most severe type of spinal cord injury; a gap following transection resulted in the loss of all motor and sensory function. In the sub-acute and chronic stages, cavities and a dense glial scar deposited by CSPGs were present in the injury site that inhibited axonal regeneration. Thus, a complete spinal cord transection model that exhibits clear and definite results to confirm axon regeneration is more appropriate for this type of study [39, 44].

### High-dose ChABC effectively digested the CSPGs in sub-acute SCI

In the present study, the high-dose ChABC treatment increased the levels of digested CSPGs compared to the control group at 4 and 10 weeks post-operation. Although ChABC may lose its enzymatic activity at body temperature [35, 36], the enzymatic activity of ChABC can persist at least ten days after a single injection into the brain of an injured rat [45]. However, a single injection of low-dose ChABC may not be sufficient to digest the CSPGs throughout the experimental period. In this study, C4S, C6S and 2B6 immunoreactivity were still detected 10 weeks after transection. These results suggested that a single injection of high-dose ChABC in sub-acute SCI could maintain the ChABC enzymatic activity throughout the experimental period, and most of the CSPGs were digested. Some C4S and 2B6 immunoreactivity was also detected in the lesion site of the transection-only rats. We presume that some endogenous matrix metalloproteinases (MMPs) and a disintegrin and metalloproteinase with thrombospondin motifs (ADAMTS) were expressed in the lesion sites and contributed to the digestion of the CSPGs [46, 47].

### Tissue preservation mediated by high-dose ChABC treatment

In the chronic stage of complete spinal cord transection, both the proximal and distal stumps of the spinal cord shrank away from the lesion site, which was filled with dense glial scar tissue [16, 24, 48–51]. These pathological phenomena were also observed in our study; the spinal cord tissue atrophied, and huge cysts formed in the bilateral stump of injured spinal cord (Fig 4D) at 10 weeks after transection. After the high-dose ChABC treatment, glial scars were also observed in the lesion site but the spinal cord tissue was preserved and more intact; the stumps did not shrink significantly, resulting in the formation of a smaller cavity (Fig 4E and 4F) compared to the control group. The glial scar components may have been altered by ChABC and resulted in chondroitin sulfate side chains dissociating from the core protein. Thus, we suggested that the ChABC-treated injured cords conserved their axons and other spinal cord tissue components and maintained the structure of the spinal cord lesion site and spinal cord stump. Therefore, ChABC may slow the process of secondary damage by decreasing cyst formation in the injured spinal cord.

### High-dose ChABC treatment promotes axonal outgrowth, regeneration and functional recovery

Various studies have demonstrated that a local application of ChABC in SCI enhances axonal sprouting/regeneration and improves functional recovery [15, 16, 21, 41, 43, 52]. The degradation of CSPGs by ChABC creates a more permissive environment for both ascending and descending axon regeneration. Our results showed that the intraparenchymal injection of high-dose ChABC in the rostral and distal penumbra sites increased the number of β-III tubulin-positive axon fibers that sprouted into the glial scar. The outgrown axons were present in the lesion site at least 10 weeks after transection, suggesting that intraparenchymal delivery of high-dose ChABC promoted and prolonged β-III tubulin-positive axon outgrowth and expression in the chronic stage of SCI. Although some β-III tubulin-positive axons were also expressed in the control group at 4 weeks after transection, very few of them passed through the dense glial scar epicenter. In addition, the scar tissue after spinal cord injury can be classified into glial and fibrotic components. The fibrotic scar that comprises an excess deposition of extracellular matrix molecules is produced by meningeal cells, which traditionally have been thought to be the main cell type [53], and perivascular fibroblasts [54]. Previous studies discovered that neurons can grow over the astrocytes but avoid fibroblasts in the co-culture system, indicating that fibroblasts are more inhibitory and less permissive to axon outgrowth [53]. Our results also exhibited the same phenomenon. Axons could pass through the GFAP+ astrocyte boundary but not the epicenter of the lesion site that represented the dense fibrotic scar [33]. Fibrotic scar formation requires dermatan sulfate (DS), which is a heterogeneous GAG and may also participate in the function of transforming growth factor beta (TGF-β) in CS upregulation in the glial scar [33, 55]. Previous studies also demonstrated that the administration of ChABC completely inhibited fibrotic scar formation in mouse brain lesions and promoted axonal regeneration [33, 56]. Consistent with previous reports, these axons could outgrow across the glial and fibrotic scar in the lesion site after the high-dose ChABC treatment.

Furthermore, the retrograde fluorescent FG tracing showed that there were no detectable FG-positive cells in the spinal cord or brainstem in the control groups (Figs 6 and 7). In the ChABC-treated groups, FG-positive neurons were clearly identified within the spinal cord rostral stump, cuneate nucleus, gracile nucleus, reticular formation, parvicellular reticular nucleus, olivary body (involved in the rubro-olivary tract, which is part of the rubrospinal tract), and medial longitudinal fasciculus. In longitudinal sections, it is difficult to precisely identify the descending tract in which the FG-positive neurons are located, but in the coronal brainstem sections, we could define the descending tract that was rewired after ChABC treatment. The rubrospinal tract is one of several major motor control pathways that are widely used in regeneration research [57–59]. The vestibulospinal tract, which arises from the medial longitudinal fasciculus, has been shown to be involved in locomotion and the control of hind limb extension during standing [60]. The motor functions might also be supported by other pathways, such as the corticospinal tract [60]. However, there were no FG-positive neurons detected in the red nucleus and motor cortex. Nonetheless, accessory nuclei also participated in the rubrospinal tract, such as the reticular formation and olivary bodies that were labeled by FG, which implied that the high-dose ChABC treatment at least partially reconnected the rubrospinal tract. These results may explain why there was only a partial improvement in the BBB open field locomotor score and in functional recovery. Despite the high levels of digested CSPG components and axonal outgrowth in the lesion site, there was only a slight improvement in the ChABC-treated groups compared to the control group in the sub-acute phase of spinal cord injury. Because β-III tubulin has been referred to as a marker of axonal regeneration [27], there were a large number of β-III tubulin-positive axons in the lesion site after ChABC treatment that may have indicated axonal sprouting and neuronal plasticity of both the injured and spared tracts following SCI [61]. However, only a few β-III tubulin-positive axons of the descending tracts were reconnected to the distal stump; most of the sprouting axons did not establish functional connections, indicating that there were too few reconnected axons to improve the functional connections [43].

### Neuronal plasticity and functional recovery

Many studies have demonstrated that ChABC administration may improve axonal plasticity in the injured spinal cord and allow neurite reconnections that will promote functional recovery [15, 61, 62]. However, it has been reported that the ChABC-mediated degradation of CSPGs does not enhance the plastic state of the spinal cord circuitry to improve functionality [63]. In this study, hind limb locomotion displayed a partial functional recovery after the high-dose ChABC treatment in the sub-acute stage of spinal cord injury, but only slight movements of the ankle, knee and hip were exhibited. The animals did not display an obvious ability to bear weight on the hind limbs at 8 and 10 weeks after spinal cord transection, suggesting that the functional reconnections were not completely established. Thus, it is critically important to enhance the process of spontaneous plasticity that occurs after SCI to enhance useful functional recovery after injury. It has been suggested that a single administration of ChABC may not be sufficient to promote functional plasticity following severe SCI [45], and a large number of studies have shown that a combination of ChABC with other interventions, such as neurotrophic factors, anti-myelin inhibitors agents, peripheral nerve grafts, biomaterials and cellular transplantation, have synergistic effects for axonal regeneration [23, 38, 49, 64, 65]. In our previous studies, we demonstrated that a combination of ChABC and olfactory mucosa progenitor cells significantly promoted axonal re-growth across the lesion site and improved the consistency of locomotion in acute SCI rats [49]. These studies indicated that multifactor strategies provide the best opportunity for plastic, functional axonal regeneration after sub-acute SCI.

## Conclusion

Our results suggest that the application of high-dose ChABC in the sub-acute stage of SCI can promote long-lasting digestion of CSPGs in the lesion site, decrease the extent and area of the lesion and decrease cyst formation in the spinal cord tissue. There was no subarachnoid hemorrhage or morphological damage following intraparenchymal injection of high-dose ChABC. The high-dose ChABC treatment significantly promoted axonal regrowth and partial functional locomotor recovery in the sub-acute stage of severe SCI, which will be important for any future clinical translation of the ChABC therapy. Furthermore, a combination of multiple therapeutic strategies, such as neuronal growth factors and cellular transplantation, with high-dose ChABC in the sub-acute stage of SCI, is necessary for promoting the functional plasticity of sprouting axons, but this approach requires further investigation.

## Supporting Information

S1 Fig FG-positive cells were expressed in the brainstem of the normal group. Coronal brain sections demonstrated that FG-positive cells were detectable in the red nucleus (A, magnifications in A”, A”’), medial longitudinal fasciculus (A, magnifications in A’), parvicellular reticular nucleus (B, magnifications in B’, B”), rubrospinal tract (C, magnifications in C’, C”), reticular formation (D, magnifications in D’, D”), and the cuneate and gracile nuclei (E, magnifications in E’, E”) in the normal group. Scale bars: A, B = 1000 μm; A’-B’ = 100 μm.
(TIF)
