# Supplementary figures and images for: Local Delivery of High-Dose Chondroitinase ABC in the Sub-Acute Stage Promotes Axonal Outgrowth and Functional Recovery after Complete Spinal Cord Transection
Source: PLoS One. 2015 Sep 22;10(9):e0138705. doi: 10.1371/journal.pone.0138705 (PMC4579094; doi:10.1371/journal.pone.0138705)

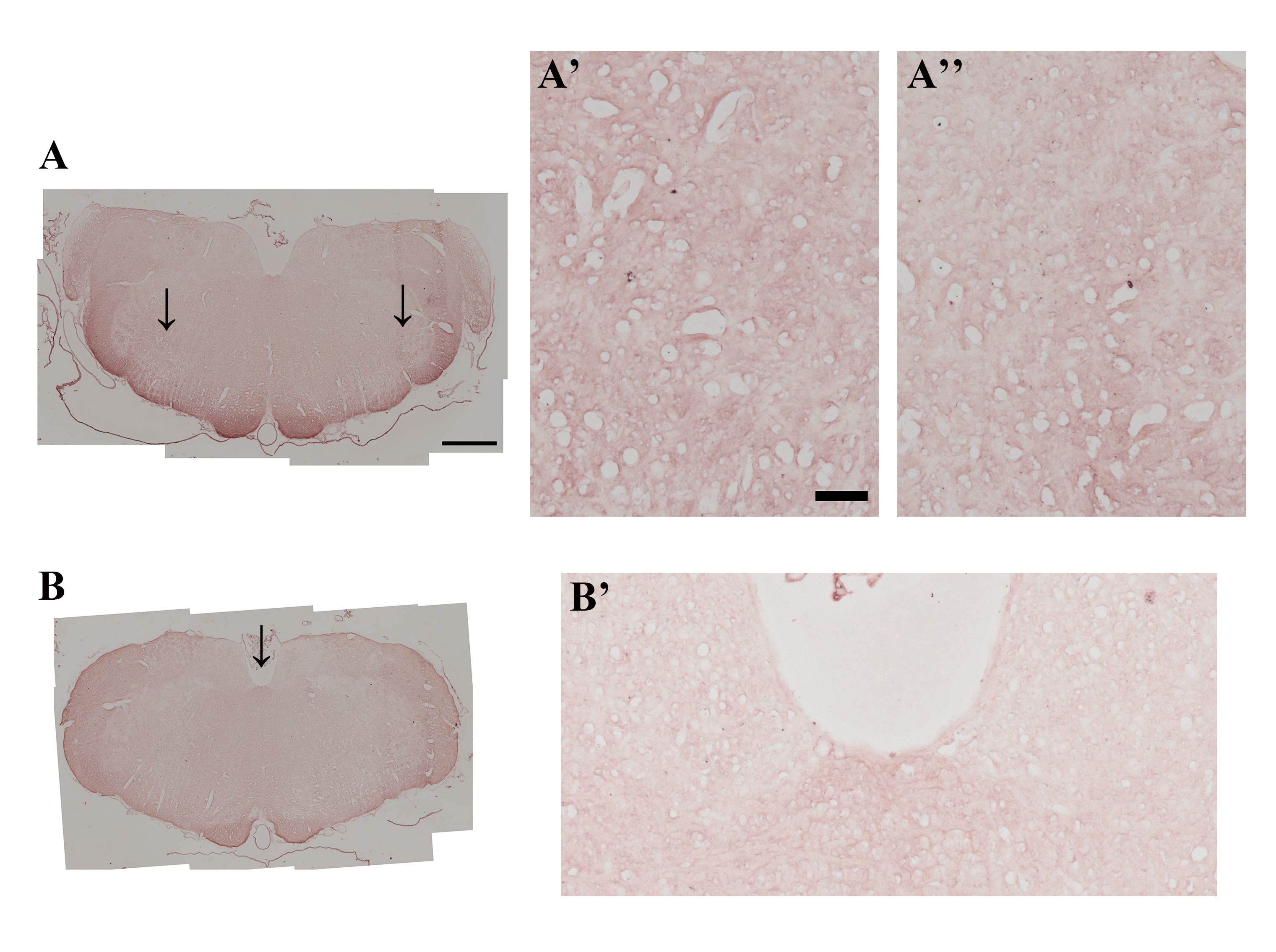

Supplement: S1 Fig — Coronal brain sections demonstrated that FG-positive cells were detectable in the red nucleus (A, magnifications in A”, A”’), medial longitudinal fasciculus (A, magnifications in A’), parvicellular reticular nucleus (B, magnifications in B’, B”), rubrospinal tract (C, magnifications in C’, C”), reticular formation (D, magnifications in D’, D”), and the cuneate and gracile nuclei (E, magnifications in E’, E”) in the normal group. Scale bars: A, B = 1000 μm; A’-B’ = 100 μm. (TIF) [file pone.0138705.s001.tif]
